# Supplementary material for: Pyruvate Dehydrogenase Kinase 1 inhibition mediated oxidative phosphorylation enhancement in cartilage promotes osteoarthritis progression
Source: BMC Musculoskelet Disord. 2023 Jul 20;24:597. doi: 10.1186/s12891-023-06585-6 (PMC10357736; doi:10.1186/s12891-023-06585-6)
Supplement: Supplementary file 1 — Additional file 1. [file 12891_2023_6585_MOESM1_ESM.zip › MF.Gsea.1653627316242/GOMF_MAP_KINASE_KINASE_KINASE_ACTIVITY.html]

Details for gene set GOMF\_MAP\_KINASE\_KINASE\_KINASE\_ACTIVITY[GSEA]

|  || Dataset | 55457\_collapsed\_to\_symbols.cls.cls #OA\_versus\_normal.cls.cls #OA\_versus\_normal\_repos |
| Phenotype | cls.cls#OA\_versus\_normal\_repos |
| Upregulated in class | normal |
| GeneSet | GOMF\_MAP\_KINASE\_KINASE\_KINASE\_ACTIVITY |
| Enrichment Score (ES) | -0.5308127 |
| Normalized Enrichment Score (NES) | -1.6640757 |
| Nominal p-value | 0.011804384 |
| FDR q-value | 0.121463805 |
| FWER p-Value | 0.954 |
Table: GSEA Results Summary

  

Fig 1: Enrichment plot: GOMF\_MAP\_KINASE\_KINASE\_KINASE\_ACTIVITY      
 Profile of the Running ES Score & Positions of GeneSet Members on the Rank Ordered List

  

| SYMBOL | TITLE | RANK IN GENE LIST | RANK METRIC SCORE | RUNNING ES | CORE ENRICHMENT || 1 | MAP3K7 | mitogen-activated protein kinase kinase kinase 7 [Source:HGNC Symbol;Acc:HGNC:6859] | 1701 | 0.399 | -0.0750 | No |
| 2 | RIPK2 | receptor interacting serine/threonine kinase 2 [Source:HGNC Symbol;Acc:HGNC:10020] | 4848 | 0.083 | -0.3074 | No |
| 3 | MAP3K13 | mitogen-activated protein kinase kinase kinase 13 [Source:HGNC Symbol;Acc:HGNC:6852] | 5728 | 0.013 | -0.3737 | No |
| 4 | RAF1 | "Raf-1 proto-oncogene, serine/threonine kinase [Source:HGNC Symbol;Acc:HGNC:9829]" | 6187 | -0.019 | -0.4065 | No |
| 5 | MAP3K2 | mitogen-activated protein kinase kinase kinase 2 [Source:HGNC Symbol;Acc:HGNC:6854] | 6976 | -0.078 | -0.4565 | No |
| 6 | MAP3K1 | mitogen-activated protein kinase kinase kinase 1 [Source:HGNC Symbol;Acc:HGNC:6848] | 7066 | -0.085 | -0.4512 | No |
| 7 | ARAF | "A-Raf proto-oncogene, serine/threonine kinase [Source:HGNC Symbol;Acc:HGNC:646]" | 8092 | -0.163 | -0.5075 | Yes |
| 8 | MAP3K14 | mitogen-activated protein kinase kinase kinase 14 [Source:HGNC Symbol;Acc:HGNC:6853] | 8100 | -0.163 | -0.4847 | Yes |
| 9 | BRAF | "B-Raf proto-oncogene, serine/threonine kinase [Source:HGNC Symbol;Acc:HGNC:1097]" | 8171 | -0.169 | -0.4660 | Yes |
| 10 | MAP3K4 | mitogen-activated protein kinase kinase kinase 4 [Source:HGNC Symbol;Acc:HGNC:6856] | 8637 | -0.203 | -0.4730 | Yes |
| 11 | MOS | "MOS proto-oncogene, serine/threonine kinase [Source:HGNC Symbol;Acc:HGNC:7199]" | 9138 | -0.243 | -0.4770 | Yes |
| 12 | MAP3K11 | mitogen-activated protein kinase kinase kinase 11 [Source:HGNC Symbol;Acc:HGNC:6850] | 9331 | -0.258 | -0.4550 | Yes |
| 13 | MAP3K9 | mitogen-activated protein kinase kinase kinase 9 [Source:HGNC Symbol;Acc:HGNC:6861] | 9712 | -0.293 | -0.4426 | Yes |
| 14 | MAP3K3 | mitogen-activated protein kinase kinase kinase 3 [Source:HGNC Symbol;Acc:HGNC:6855] | 10121 | -0.333 | -0.4265 | Yes |
| 15 | MAP3K6 | mitogen-activated protein kinase kinase kinase 6 [Source:HGNC Symbol;Acc:HGNC:6858] | 10317 | -0.353 | -0.3912 | Yes |
| 16 | TAOK2 | TAO kinase 2 [Source:HGNC Symbol;Acc:HGNC:16835] | 10718 | -0.395 | -0.3657 | Yes |
| 17 | MAP3K5 | mitogen-activated protein kinase kinase kinase 5 [Source:HGNC Symbol;Acc:HGNC:6857] | 10923 | -0.421 | -0.3212 | Yes |
| 18 | MAP3K12 | mitogen-activated protein kinase kinase kinase 12 [Source:HGNC Symbol;Acc:HGNC:6851] | 11344 | -0.478 | -0.2854 | Yes |
| 19 | MAP3K20 | mitogen-activated protein kinase kinase kinase 20 [Source:HGNC Symbol;Acc:HGNC:17797] | 11475 | -0.498 | -0.2242 | Yes |
| 20 | MAP3K10 | mitogen-activated protein kinase kinase kinase 10 [Source:HGNC Symbol;Acc:HGNC:6849] | 11753 | -0.541 | -0.1682 | Yes |
| 21 | RIPK1 | receptor interacting serine/threonine kinase 1 [Source:HGNC Symbol;Acc:HGNC:10019] | 11953 | -0.584 | -0.1000 | Yes |
| 22 | MAP3K8 | mitogen-activated protein kinase kinase kinase 8 [Source:HGNC Symbol;Acc:HGNC:6860] | 11975 | -0.589 | -0.0174 | Yes |
| 23 | EGFR | epidermal growth factor receptor [Source:HGNC Symbol;Acc:HGNC:3236] | 12109 | -0.622 | 0.0614 | Yes |
Table: GSEA details [plain text format]

  

Fig 2: GOMF\_MAP\_KINASE\_KINASE\_KINASE\_ACTIVITY      
 Blue-Pink O' Gram in the Space of the Analyzed GeneSet

  

Fig 3: GOMF\_MAP\_KINASE\_KINASE\_KINASE\_ACTIVITY: Random ES distribution      
 Gene set null distribution of ES for **GOMF\_MAP\_KINASE\_KINASE\_KINASE\_ACTIVITY**
